# Structured Lipids Engineering for Health: Novel Formulations Enriched in *n*-3 Long-Chain Polyunsaturated Fatty Acids with Potential Nutritional Benefits

**DOI:** 10.3390/metabo13101060

**Published:** 2023-10-08

**Authors:** Paula A. Lopes, Cristina M. Alfaia, José M. Pestana, José A. M. Prates

**Affiliations:** 1 CIISA—Centro de Investigação Interdisciplinar em Sanidade Animal, Faculdade de Medicina Veterinária, Pólo Universitário do Alto da Ajuda, Universidade de Lisboa, Avenida da Universidade Técnica, 1300-477 Lisbon, Portugal; cpmateus@fmv.ulisboa.pt (C.M.A.); jpestana@fmv.ulisboa.pt (J.M.P.); japrates@fmv.ulisboa.pt (J.A.M.P.); 2 Laboratório Associado para Ciência Animal e Veterinária (AL4AnimalS), Faculdade de Medicina Veterinária, Universidade de Lisboa, 1300-477 Lisbon, Portugal

**Keywords:** TAG, SL, stereospecific position, *n*-3 LCPUFA, health benefits

## Abstract

Structured lipids (SLs) offer a promising avenue for designing novel formulations enriched in *n*-3 long-chain polyunsaturated fatty acids (LCPUFAs) with potential health benefits. Triacylglycerols (TAGs), the most common fats in the human diet, are both non-toxic and chemically stable. The metabolic efficiency and digestibility of TAGs are significantly influenced by the position of fatty acids (FAs) within the glycerol backbone, with FAs at the *sn*-2 position being readily absorbed. Over the past two decades, advancements in SL research have led to the development of modified TAGs, achieved either through chemical or enzymatic processes, resulting in SLs. The ideal structure of SLs involves medium-chain FAs at the *sn*-1,3 positions and long-chain *n*-3 LCPUFAs at the *sn*-2 position of the glycerol backbone, conferring specific physicochemical and nutritional attributes. These tailored SL formulations find wide-ranging applications in the food and nutraceutical industries, showing promise for dietary support in promoting health and mitigating various diseases. In particular, SLs can be harnessed as functional oils to augment TAG metabolism, thereby impeding the development of fatty liver, countering the onset of obesity, and preventing atherosclerosis and age-related chronic diseases. In scrutinising prevailing research trajectories, this review endeavours to provide an in-depth analysis of the multifaceted advantages and repercussions associated with the synthesis of SLs. It elucidates their burgeoning potential in enhancing health and well-being across a range of demographic cohorts. Specifically, the implications of SL utilisation are discussed in the context of healthcare environments and early childhood developmental support.

## 1. Introduction

Fats and oils are defined as complex organic molecules formed by combining three fatty acids (FAs) with one molecule of glycerol. Fats with *n*-3 polyunsaturated fatty acids (PUFAs) present in fish and plants are almost exclusively triacylglycerols (TAGs), also known as triglycerides [1]. The molecular arrangement of various TAGs is a determinant factor of metabolic fate in the body, especially at the levels of digestibility, small intestine absorption, and bioavailability, and is improved for FAs allocated at the *sn*-2 position. The first mention that FAs at the *sn*-2 position are preferentially absorbed was reported by Jensen et al. [2] and subsequently in studies by Xu [3] and Hunter [4]. Following a chronological order, a pioneer characterisation of the nutritional value of different fish oils established that docosahexaenoic fatty acid (DHA, 22:6*n*-3) had a preference for the *sn*-2 position while eicosapentaenoic fatty acid (EPA, 20:5*n*-3) tendentially occupied *sn*-1 and *sn*-3 positions [5]. EPA and DHA constitute *n*-3 long-chain PUFAs (LCPUFAs) and have a range of physiological roles*,* which are linked to health or clinical benefits*,* particularly related to neurological and cardiovascular diseases [6].

Across the USA and in some European locations, the dietary consumption of marine or fishy *n*-3 LCPUFAs is far from the recommendation for human health [7]. The mean intake of DHA combined with EPA from food may vary between 88 and 226 mg/day in Europe [8], therefore concluding that the beneficial effects of *n*-3 LCPUFAs can only be achieved through supplementation [9]. In parallel, the permanent exploitation of fishery resources to obtain fish and related products is aggravating their sustainability to the limit [10,11]. Several obstacles interfere with the nutritional recommendations for *n*-3 LCPUFAs consumption, in particular, nutritional habits, apprehension regarding methyl mercury found in some fishes, and price and low stability of fishy oils included in food products. Additionally, existing stocks of wilding and farmed fish species are indeed scarce [12]. In line with this, searching for other options for *n*-3 LCPUFAs is mandatory to enhance the availability of EPA and DHA. Such an option is microalgae. Microalgae cultivation is an excellent option for auto-sustainable and eco-friendly *n*-3 LCPUFAs sources since microalgae do not demand fresh water or arable land for production, display the capability of elevated FAs deposition (over 20%), and have the ability to generate EPA and DHA oils with high purity [10]. In addition to education focused on changing dietary patterns, dietary supplements stand out as an alternative to improve human health, particularly for senior citizens. At present, a large variety of food supplements are available commercially, with different contents, degrees of purity, types, and molecular structures of *n*-3 LCPUFAs, essentially because of the small efficacy on the conversion of α-linolenic acid (ALA, 18:3*n*-3) into EPA and DHA [13,14,15]. In this regard, chemical or enzymatic synthesis of structured lipids (SLs), particularly of various molecular forms of TAGs enclosing *n*-3 LCPUFAs, has received much attention [16], yet scientific information on the bioavailability, bioaccessibility, and putative health protective effects of SLs remains elusive.

The objective of this review was to offer an in-depth examination of SLs, incorporating the most recent advancements in their synthesis. This review also aimed to elucidate the potential applications of SLs in metabolic and nutritional contexts, with particular emphasis on their relevance in healthcare settings and child development support.

## 2. Structured Lipids Engineering

SLs are TAGs chemically and/or enzymatically changed for functional, nutritional, and health properties [17]. The outcome is TAGs with different combinations of FA chain lengths on the glycerol backbone. The engineering of SLs with a specific chemical structure allows for the modulation of TAGs behaviour and has received much recognition [4].

The classification of FA arrangements on the glycerol backbone is generally segmented into four distinct types: Medium-Long-Medium (MLM), Medium-Medium-Long (MML), Long-Medium-Long (LML), and Long-Long-Medium (LLM) [18]. Of these, MLM-type SLs are of particular interest, comprising medium-chain fatty acids (MCFAs) esterified at the *sn*-1 and *sn*-3 positions and a long-chain fatty acid (LCFA) at the *sn*-2 position of the glycerol backbone. This particular arrangement has been identified as the most favourable for efficient absorption via the intestinal mucosa [19,20].

Notably, MLM-type SLs exhibit unique physiological properties compared to long-chain triacylglycerols (LCT), the standard TAG structure commonly found in most fats and oils. This distinct physiological behaviour can be attributed to the presence of both MCFAs and LCFAs in the same TAG molecule [21]. MCFAs are rapidly absorbed into the portal circulation, thereby serving as quick sources of energy. In addition, LCFAs follow the more conventional absorption pathway involving micelle formation and chylomicron assembly. The coexistence of both MCFAs and LCFAs in MLM-type SLs, therefore, creates a lipid source that offers both quick and sustained energy release, thereby offering potential metabolic advantages.

The synthesis and production of MLM-type SLs have been the subject of numerous studies exploring the feasibility of deriving these specialised lipids from various plant oils [19,22,23,24,25,26,27,28,29,30,31,32,33,34]. Given the distinct advantages associated with MLM-type SLs, considerable effort has been invested in optimising the enzymatic or chemical routes for their synthesis. This has led to a variety of methods to produce MLM-type SLs, such as interesterification and enzymatic catalysis, each with its own set of advantages and limitations.

Such insights hold significant implications not only for the field of nutrition science but also in animal science and food safety. The distinctive properties of MLM-type SLs make them prime candidates for specialised dietary formulations in animal feeds and functional foods designed for optimised human nutrition.

### 2.1. Scale-Up Synthesis and Stereospecific Position of FAs in SLs

SLs are produced using two types of catalysts: chemical or enzymatic interesterification. Comparing both processes, chemical interesterification is a low-priced method and requires less time reaction than enzymatic interesterification [35]. However, during chemical interesterification, some complications may happen with stereospecificity, except if lower temperatures are applied to prevent the generation of random re-arrangements of FAs [36].

SLs can be produced via different reaction types, described as follows:

- Interesterification reactions are defined by the exchange of fatty acyl groups between two or more TAG molecules [37]. The reaction begins with TAG hydrolysis into free fatty acids (FFA) followed by re-esterification of the FFA on the glycerol backbone. Numerous commercial fats apply this method, the most common being Betapol (Lipid Nutrition) and Salatrim (short and acyltriacylglycerol molecules) [36]. Betapol is illustrative of human milk fat (HMF) analogues [38], whereas Salatrim encloses low energy value (~5 kcal/g) synthesised by short-chain FAs, therefore supplying lower calories than LCFAs;
- Acidolysis reactions are defined by the transfer of the acyl group between an acid and an ester [39]. Previous reports have successfully applied enzymatic acidolysis for the synthesis of new SLs with a high amount of long-chain PUFAs at the *sn*-2 position of TAGs [40,41,42];
- Alcoholysis reactions are defined by the exchange of the alkoxy group between an alcohol and an ester, like glycerol (glycerolysis) or ethanol (ethanolysis) [39].

**Figure 1:**
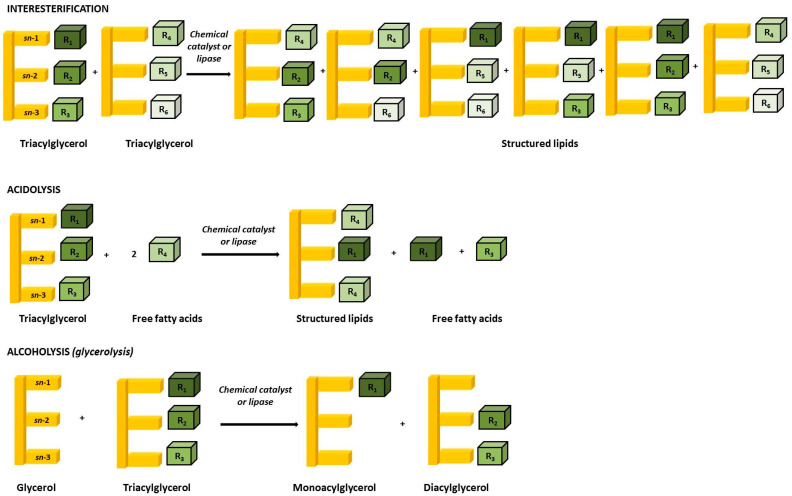
Schematic overview of SLs synthesis representing interesterification, acidolysis, and alcoholysis (glycerolysis) reactions.

These schematic reactions are illustrated in Figure 1.

The enzymatic synthesis of SLs makes use of biocatalysts, lipase, and phospholipase enzymes to modify fats and oils [43] because of their high selectivity and regiospecificity [36,44,45]. For instance, *sn*-1,3-specific lipases have favouritism for the acyl ester bonds at the first and third positions of the acylglycerols enclosing FAs at these locations with no modification of FAs at the *sn*-2 position [18,45]. In contrast, non-specific lipases do not demonstrate different specificity about the position of the acyl ester group on the glycerol backbone. It should be noted that enzymatic reactions are driven by soft temperatures, up to 35 °C [46,47], with negligible loss of prime attributes of temperature-sensitive substrates and products (in particular, SLs) [18]. In addition, enzymes constitute an environmentally friendly solution because they reduce energy demand as well as the need for hazardous reagents [18].

The selection of immobilised enzymes grants recovery and re-use of enzymes over time, decreasing the financial burden, which constitutes an additional advantage of the enzymatic processes [18]. To exemplify, commercial enzymatic products are Lipozyme RM IM (*Rhizomucor miehei* lipase immobilised on a macroporous anion exchange resin, *sn*-1,3 specific enzyme), Lipozyme TL IM (*Thermomyces lanuginosus* lipase immobilised on silica gel, *sn*-1,3 specific enzyme) and Novozym 435 (*Candida antarctica* lipase B immobilised on a macroporous acrylic resin and *Candida rugosa*, non-specific enzymes) [18].

The allocation of FAs at the *sn*-2 position might be confirmed through thin-layer chromatography (TLC) procedures for lipids separation, as reported by Álvarez and Akoh and changed by Luddy and colleagues for the pancreatic lipase-catalysed *sn*-2 positional analysis of blending oils [48,49]. 2-Oleyglycerol was run as standard at the same time as the sample for 2-monoacylglycerol (2-MAG*)* band identification. Next, the 2-MAG band was scrapped off and changed to fatty acid methyl esters (FAME). The FAs profile at the *sn*-2 position was determined by gas chromatography (GC) [48]. As an alternative, Guil-Guerrero and colleagues reported the positional distribution of DHA at the *sn*-2 position within the TAGs molecular structure by ^13^C-nuclear magnetic resonance (^13^C-NMR) [50]. The information on the preparation methods of SLs is summarised in Table 1.

Oleogelation, a recent technique for obtaining SLs, is also considered a vehicle of FA delivery. The type of vegetable oil involved in the formation of the oleogel must be well-chosen since recent research demonstrates that its composition, the carbon chain length of the FAs, and the unsaturation level of FAs are factors influencing the oleogel properties and its behaviour in food matrices [60,61,62].

### 2.2. Clinical Studies and Prospective Outcomes of SLs

The health benefits of SLs in human and animal models are described in detail and in chronological order in Table 2. As early as 1995, Sandström et al. described that the administration of an SL emulsion enclosing medium- and long-chain FAs, esterified in a random way to glycerol in a TAG molecular structure enhanced whole-body fat oxidation in postoperative patients [63]. Corroborating these outcomes, patients who underwent abdominal surgery and received an enteral diet comprising fish oil/medium-chain TAGs structured lipids ameliorated hepatic and renal function, as well as immunity, and presented considerably reduced eicosanoids from peripheral blood mononuclear cells [64]. In the meantime, Bellantone et al. published the first report in which structured TAGs were given to postoperative patients to assess safety, tolerance, and efficacy [65]. The major finding was that clinical parameters were comparable between structured TAGs and long-chain TAGs [65].

Except for the above-mentioned studies with humans, the literature on this topic relies mostly on the utilisation of experimental animals, permitting extrapolation to humans. SLs were applied as functional oils to suppress high fat-induced obesity in mice by diminishing plasma TAGs [66]. In addition, DHA-enriched SLs-DAG diminished body fat and liver steatosis in fatty models by ameliorating hepatic FAs as well as related gene expression [67]. Additionally, rice bran oil with ALA from linseed oil (LSO) and EPA and DHA from fish oil at TAG *sn*-1 and *sn*-3 positions showed hypocholesterolaemic and hypolipidaemic properties [68,69]. The same was reported by Sharma and Lokesh for SLs from groundnut oil and *n*-3 FAs from LSO [70]. In general, these outcomes sustain the evidence that SLs beneficially diminished blood lipids and lipid deposition in animals fed atherogenic diets [69,71]. The hypotheses underlying Nagata et al. [72,73] studies were based on (1) lipid emulsions of very pure SLs enclosing medium-chain FAs allocated at the *sn*-1 and *sn*-3 positions and linoleic acid (LA, 18:2*n*-6) allocated at the *sn*-2 position are quickly hydrolysed; (2) diets with extremely pure LML type ameliorate blood and hepatic lipids; and (3) whether MLM-type lipids could act as the favourite pancreatic substrate and be a contributor for energy supply.

In cholesterol-rich blood, weak and deformed red blood cells from rats were, in part, counteracted by SLs, specifically EPA and DHA-rich mustard oil, by ameliorating blood counts and histology, as well as by reversing hypercholesterolemia [74,75]. Conversely, Kim et al. demonstrated that the dietary effects of sesame oil-based MLM-type SLs on plasma lipaemia and cardiovascular function were undistinguished from those of original sesame oil (LCT) but promoted tachycardia in hypertensive rats [21,53].

Using rat models for standard and mal absorption, Straarup and Høy reported that the maximum absorption of a structured fat is highly dependent on regiospecific structure [76]. As so, SLs enclosing medium- and long-chain FAs fed to post-weaning piglets impacted positively on nitrogen digestibility and faecal fat as well as on long-chain FA deposition [77].

Yet, reports thus far with structured TAGs containing DHA esterified at the *sn*-2 position are very limited. Our research team has conducted studies using only healthy animal models [13,14]. It was hypothesised that the incorporation of DHA across adipose tissues will be higher when it is ingested as TAGs structured at the *sn*-2 position [14]. The structured *sn*-2 position DHA-containing TAGs improved blood lipids and FA incorporation, in particular EPA and DHA in the liver, erythrocytes, and brain, relative to commercial fish oils, thus improving the health benefits of DHA due to its higher bioavailability [13]. However, results do not document augmentation of the anti-adipogenic effect of DHA structured at the *sn*-2 position of TAGs nor suggest that more DHA relative to EPA could reduce the accumulation of body fat in hamsters. This might be a direct consequence of using normal-weight hamsters [14].

Another aspect is related to the impact of SLs on immune function by improving cell phagocytosis, which is a paramount phenomenon for host defence against pathogens. Kew et al. studied in vitro the effects of structured TAGs enriched in EPA and DHA on splenocyte FA profiles and leucocyte phagocytosis [78]. Conversely to DHA, a clear effect of EPA was reported at the *sn*-2 position of TAGs, rather than at the *sn*-1 or *sn*-3 positions, on its deposition in cell phospholipids and phagocytic cell action in a dosage-dependent way [78].

**Table 2:** Health benefits of SLs using different experimental models.

SL Type	Beneficial Health Effects	Experimental Model	References
Medium-chain triacylglycerols (MCTs) and LCTs	Increased fat oxidation in postoperative patients	Human	Sandström et al. [63]
Fish oil/MCTs	Ameliorated immunity and hepatic and renal function Reduced eicosanoids from peripheral blood mononuclear cells	Human	Swails et al. [64]
SLs containing EPA, DHA, and caprylic acid	Diminished cholesterol and TAGs	Mouse	Lee et al. [71]
Rapeseed oil-based MLM-type	Ameliorated the FA hydrolysis as well as absorption	Rat	Straarup and Høy [76]
LML-type MLM-type	MLM-type quickly hydrolysed and impacted on energy supply LML ameliorated blood and hepatic lipids	Rat	Nagata et al. [72]
MLM-type LMM-type	Both types of SLs diminished blood lipids and cholesterol	Rat	Nagata et al. [73]
Rapeseed oil-based MLM-type	Ameliorated fat in faeces and nitrogen digestibility Greater accumulation of LCFAs	*Post*-weaning piglet	Straarup et al. [77]
Sesame oil-based MLM-type	Without effects on the cardiovascular system	Spontaneously hypertensive rat	Kim et al. [53]
DHA-enriched structured-DAG	Ameliorated FAs and cholesterol	Mouse	Kim et al. [67]
Sesame oil-based MLM-type	Damaged cardiovascular system and produced tachycardia	Spontaneously hypertensive rat	Kim et al. [21]
SLs with cod liver oil, SLs with linseed oil	Hypolipidaemic and hypocholesterolaemic properties	Rat	Chopra and Sambaiah [68]
MCTs-containing mustard oil PUFA-containing mustard oil	SLs counteracted thrombocyte aggregation and showed hypercholesterolaemic effects	Hypercholesterolemic rat	Sengupta and Ghosh [74]
MCTs-rich mustard oil PUFA-rich mustard oil	Reduced deleterious influence of cholesterol in red blood cell membranes	Rat	Sengupta and Ghosh [75]
SLs containing short-chain fatty acids (SCFAs)	Diminished TAGs	Mouse	Cao et al. [66]
SLs with sunflower and SLs with soybean oil with ethyl behenate	Diminished lipaemia and lipid deposition	Rat and rabbit	Kanjilal et al. [69]
SLs with groundnut oil, SLs with linseed oil	Diminished LDL cholesterol and TAGs	Rat	Sharma and Lokesh [70]
*sn*-2 position DHA- containing TAGs	Improved blood lipids and EPA and DHA deposition in the liver, erythrocytes, and brain	Hamster	Bandarra et al. [13]
*sn*-2 position DHA- containing TAGs	No anti-adipogenic effect of DHA	Hamster	Lopes et al. [14]

On the topic of exercise performance, the few studies performed so far have not used SLs but MCTs based on the possibility of sparing glycogen during exercise through rapid oxidation of the medium-chain FAs for fuel [16]. After digestion and absorption, the medium-chain FAs turn into fuel, reaching hepatic cells and providing energy through mitochondrial oxidation, increasing hypothetical endurance. Another positive outcome of the use of MCTs in place of carbohydrates would be to avoid insulin rise and possible hypoglycaemia during intensive exercise. Although these hypotheses have been described as a putative mechanism for a metabolic benefit for medium-chain FAs resulting from MCTs or SLs, there is no evidence to support their validity. MCTs were demonstrated in one study to blunt the increased insulin levels occurring after ingestion of an isocaloric amount of carbohydrates, but during exercise, carbohydrates were preferentially oxidised compared to MCTs [79]. Ivy et al. did not show a reduction in insulin levels when MCTs replaced carbohydrates [80]. These studies do not provide support for the use of MCTs in the enhancement of exercise performance. The direct extrapolation of this conclusion to SLs is adequate because the unique fuel provided by SLs is the medium-chain FA component. Medium-chain FAs from SLs follow the same digestion and absorption process as medium-chain FAs from MCTs [81].

### 2.3. Human Milk Fat Analogues for Infants

Human milk fat (HMF) is the second largest constituent of breast milk by concentration (3–5% in mature milk) and donates approximately half of the energy provided to infants through diet [82]. In terms of FA characterisation, HMF is enriched in essential fatty acids (EFAs), like ALA and LA, and their derivatives, like LCPUFAs, DHA, and arachidonic acid (ARA, 20:4*n*-6), which exist in human milk at residual levels (for each, <1%) [83,84]. During infant development, the bioavailability of essential FAs and LCPUFAs are vital for brain growth and development, motor ability, cognitive skills, neurological reflexes, and sensory functions, being critical for DHA and its role regarding memory and visual skills [85,86]. In line with this need, the majority of infant formulations available on the market are supplemented with ARA and DHA, especially for preterm newborns [38]. However, several studies described that even when traditional formulations contain considerable contents of LA and ALA (the precursors of ARA and DHA endogenous synthesis, respectively), these formulations were unable to convey postnatal LCPUFA acceptable levels both in the plasma and erythrocytes of infants fed human breast milk [87,88]. Although debatable, some authors reported that elongation–desaturation enzymes are not fully active to completely desaturate and elongate LA and ALA during the initial stages of life [89]. Most importantly, commercial infant formulations available in the market vary at the positional distribution of the most beneficial FAs on TAG molecular structure in comparison to HMF [52,90]. For instance, ARA (approximately 45%) and DHA (approximately 60%) are commonly located at the *sn*-2 position in human milk fat, while in infant formulations, their distribution is nearly alike across TAG *sn*-positions [52]. DHA- and ARA-rich single-cell oils (DHASCO and ARASCO, correspondingly) do not display positional specificity, with FAs positioned practically indistinctly at all *sn*-positions [91]. Moreover, the FA position of TAGs has a key role regarding absorption, distribution, and fat metabolism in infants [92,93]. Christensen and Høy reported that newborn rats fed oils enclosing ARA and DHA at the *sn*-2 position of TAGs showed increased values of ARA and DHA in the brain in comparison to newborn rats fed with oils enclosing the same FAs randomly allocated [94]. ARA and DHA located at TAG *sn*-1 and *sn*-3 positions promote resistance to pancreatic lipase, and consequently, small absorption of these FAs is expected to occur [95].

All in all, HMF analogues are described as SLs comparable to human milk fat in what concerns FAs profile and distribution developed for application in infant formulations [18]. Betapol was the first SL commercially developed as an HMF analogue, even if the LCPUFA content was still deficient [18,96,97]. That is the reason novel SLs reinforced with LCPUFAs have been produced for successful infant growth and development by applying distinct oils in conjugation and with reasonable contents of DHA located at the *sn*-2 position [54,55,56,97,98,99]. Several researchers developed HMF analogues adequate for infant formulations that successfully convey ARA, DHA, and other LCPUFAs for pregnant and vegan people [100,101]. A few years ago, Álvarez and Akoh tested with success an infant formulation fat analogue with great contents of ARA and DHA at the *sn*-2 position [48,52]. Information about the methods of synthesis and nutritional benefits of human milk fat analogues is presented in Table 3.

## 3. Conclusions

In conclusion, the health implications of specific FAs are influenced by both their molecular structures and their forms of administration. TAGs, abundant in the human diet and recognised for their safety and chemical stability, serve as a primary carrier of these FAs. The positional arrangement of FAs, particularly at the *sn*-2 location in TAGs, is a critical factor affecting their bioaccessibility, bioavailability, physiological properties, and metabolic pathways in vivo.

SLs are TAG molecules modified either chemically or enzymatically to rearrange or incorporate new fatty acids. Notably, the physiological effects of SLs seem to be more dependent on the length of the FAs chain rather than intricate structural details. The stability of these beneficial structured TAGs can be significantly enhanced by the incorporation of suitable antioxidants, as the ingestion of oxidised lipids may detrimentally affect a host of physiological markers, such as lipid metabolism, oxidative stress, and vascular function.

Enzymatic interesterification is frequently employed for lipid structuring, especially in the synthesis of MLM SLs. In this arrangement, medium-chain FAs are strategically positioned at the *sn*-1 and *sn*-3 sites, with a long-chain FA located at the *sn*-2 position. This configuration is deemed ideal for maximising the health-promoting effects of FAs. Moreover, SLs may have future applications as analogues to human milk fat in infant formulas, especially when enriched with preformed DHA for added nutritional benefit.

However, caution is advised in the selection of antioxidants for SL-based products, as certain compounds like α-tocopherol, β-carotene, and soy isoflavones may exhibit pro-oxidant activity under specific conditions. Thorough experimental validation is therefore essential before incorporating these antioxidants into SL-based functional products.

Overall, the thoughtful design and application of SLs present a promising avenue for leveraging the health-promoting attributes of specific FAs. The strategic positioning of FAs in the TAG structure, coupled with the use of appropriate antioxidants, can markedly amplify the physiological benefits of SLs. This makes them viable candidates for various health and nutritional applications, including functional oils and the development of specialised infant formulas.

## 4. Take-Home Message

- The engineering of SLs with precise TAG configurations allows for the fine-tuning of their bioavailability and bioactivity, thereby optimising their nutritional and physiological benefits;
- Enzymatic interesterification techniques offer a promising approach for the targeted synthesis of SLs, particularly those with MLM arrangements that are optimal for health benefits;
- SLs represent a frontier in nutritional science, with potential applications extending beyond general health, targeting specific needs in healthcare settings and child development.

## Figures and Tables

**Table 1:** Brief description on the preparation methods of SLs.

Type of Method	Reaction	Nutritional Applications	References
Chemical	Interesterification	Shortenings and *trans*-FAs free margarine	Rousseau and Marangoni [51]
		Infant formulation (e.g., Betapol)	Farfán et al. [36]
Enzymatic	Interesterification applying *sn*-1,3 specific lipases	Infant formulation enriched with ARA and DHA	Álvarez and Akoh [48,52]
		Reduced calorie fat (e.g., Salatrim)	Farfán et al. [36]
Chemical	Acidolysis	Bakery products	Rousseau and Marangoni [51]
		MLM synthesis	Kim et al. [21,53]
Enzymatic	Acidolysis applying *sn*-1,3 specific lipases	Infant formulation	Sørensen et al. [54], Li et al. [55]
		Infant formulation enriched with DHA	Pande et al. [56]
Chemical	Alcoholysis	Surfactants, emulsifiers	Feltes et al. [57]
Enzymatic	Ethanolysis applying *sn*-1,3 specific lipases	Emulsifiers MAG	Wang et al. [58]
	Glycerolysis applying *sn*-1,3 specific lipases	Emulsifiers DAG oil	Flickinger and Matsuo [59]

**Table 3:** Effects of different methods of SLs synthesis on human milk fat analogues.

SLs Synthesis Methods	Formulation Tested	Beneficial Health Effects	References
Acidolysis	Butterfat plus soybean oil and rapeseed oil FAs	Reduced oxidative stability Potential application as infant formulation	Sørensen et al. [54]
Interesterification	18:4*n*-3 soybean oil plus tripalmitin	*n*-3 FA beneficial health effects Potential application as infant formulation	Teichert and Akoh [98]
Interesterification (step 1) Acidolysis (step 2)	Step 1: 18:4*n*-3 soybean oil plus tripalmitin = SLs Step 2: SLs plus 18:3*n*-6 or DHA	Considerable contents of 18:3*n*-6 and DHA Potential application as infant formulation	Teichert and Akoh [99]
Acidolysis	Palm olein plus DHASCO-FFA and ARASCO-FFA	Increased contents of ARA and DHA at the *sn*-2 position Potential application as infant formulation	Nagachinta and Akoh [100]
Interesterification (for both steps)	Step 1: hazelnut oil plus 16:0 ethyl ester = 16:0-rich SLs Step 2: 16:0-rich SLs plus ARASCO and DHASCO	Health benefits associated with ARA and DHA Potential application as infant formulation	Turan et al. [101]
Interesterification plus acidolysis	Extra virgin olive oil plus tripalmitin plus ARASCO-FFA plus DHASCO-FFA	Reasonable content of DHA at the *sn*-2 position Potential application as infant formulation	Pande et al. [97]
Acidolysis	Tripalmitin plus extra virgin olive oil-FFA plus DHASCO-FFA	Considerable content of DHA at the *sn*-2 position Potential application as infant formulation	Pande et al. [56]
Acidolysis	Refined olive oil plus 16:0 + DHA	Reasonable content of DHA at the *sn*-2 position Potential application as infant formulation	Li et al. [55]
Interesterification	Synthesis of high *sn*-2 DHA and ARA oils through DHASCO and ARASCO	High contents of DHA and ARA at the *sn*-2 position Potential application as infant formulation	Álvarez and Akoh [48]
Interesterification (for step 1)	Step 1: *sn*-2 16:0 SLs plus capric acid = SL_CA_ Step 2: Blending SL_CA_ with canola oil, corn oil, high *sn*-2 DHA, and high *sn*-2 ARA	DHA and ARA predominantly at the *sn*-2 position Potential application as infant formulation enriched with medium chain FAs, ARA, and DHA	Álvarez and Akoh [52]

## Abbreviations

ARASCO—arachidonic acid single cell oil; DHASCO—docosahexaenoic acid single cell oil; LCT—long-chain triacylglycerols; LLM—long, long, medium-chain triacylglycerols; LML—long, medium, long-chain triacylglycerols; MCFAs—medium chain fatty acids; MCTs—medium-chain triacylglycerols; MLM—medium, long, medium-chain triacylglycerols; MML—medium, medium, long-chain triacylglycerols; SCFAs—short chain fatty acids; SLs—structured lipids; SLs-DAG—structured lipids-diacylglycerol; *sn*—stereospecific numbering; TAGs—triacylglycerols.
